# Simulating the Spread of Foot-and-Mouth Disease in Densely Populated Areas as Part of Contingency Plans to Establish the Best Control Options

**DOI:** 10.3390/pathogens14090933

**Published:** 2025-09-16

**Authors:** Silvia Bellini, Alessandra Scaburri, Marco Tironi, Veronica Cappa, Alessandro Mannelli, Giovanni Loris Alborali

**Affiliations:** 1 Istituto Zooprofilattico Sperimentale della Lombardia ed Emilia-Romagna, Via A. Bianchi 9, 25124 Brescia, Italy; alessandra.scaburri@izsler.it (A.S.); marco.tironi@izsler.it (M.T.); veronica.cappa@izsler.it (V.C.); giovanni.alborali@izsler.it (G.L.A.); 2 Department of Veterinary Sciences, University of Turin, Largo P. Braccini 2, 10095 Grugliasco, Italy; alessandro.mannelli@unito.it

**Keywords:** foot-and-mouth disease, FMD, densely populated livestock areas, DPLAs, contingency plan, preparedness, disease control

## Abstract

Foot-and-mouth disease (FMD) is a highly contagious disease of livestock caused by the FMD virus (FMDV). It is not dangerous to humans but can cause severe disruption to the farming sector and hampers trade in animals and animal products. Given the characteristic of transmissibility of the virus, the legislation in force in the European Union requires that some control activities be initiated in peacetime: the so-called “emergency preparedness”. As part of a research project on FMD, a dynamic transmission model was developed to test the effectiveness of the main control strategies in different livestock settings in Italy. This manuscript focuses on the control of the disease in densely populated livestock areas (DPLAs). Reduction in farm density was simulated to identify a threshold density compatible with disease control in the study area and to understand whether this was acceptable in terms of the sustainability of the livestock production system. Considering that in some municipalities the density of animals greatly exceeded the identified threshold, we adopted an original risk-based approach aimed at identifying farms which were most likely to play a central role in FMDV transmission. This approach has proven to be the most effective in controlling the spread of FMDV and can be proposed for practical applications where limited information on contacts between farms is available.

## 1. Introduction

Foot-and-mouth disease (FMD) is a highly contagious viral disease of cloven-hoofed domestic and wild animals. The virus responsible for FMD is an aphthovirus of the Picornaviridae family (FMDV). It is highly infectious and can spread by direct contact between infected and susceptible animals, or contact with contaminated farming equipment, products of animal origin, vehicles, clothing, and feed [1].

In the first quarter of 2025, two distinct FMDV incursions were reported in the European Union (EU). The first case occurred in Germany on 10 January 2025, and this event ended with a single outbreak on a water buffalo farm located near Berlin. The second incursion was detected in Hungary on 3 March 2025, near the Danube River and the border with Slovakia, where the disease was confirmed on 20 March 2025. Since then, in just over a month, eleven outbreaks have been identified in this border area, five in Hungary and six in Slovakia [2]. These cases represent the first FMD outbreaks in the EU since 2011, when FMD occurred in the Burgas Province of Bulgaria.

FMD is a major constraint to international trade and countries currently free of FMD take every precaution to prevent its entry. Its containment demands considerable efforts in strict monitoring, quarantines, culling of both infected and potentially infected in-contact animals. Apart from the recent occurrences in Germany, Hungary and Slovakia, the disease has been eradicated from the territory of the EU and the World Organisation for Animal Health (WOAH) has recognised EU Member States as “free of FMD without vaccination”. However, the disease has been reported in some countries bordering the EU, which poses a serious threat to the EU Member States, as is also clearly confirmed by the recent FMD incursions reported in 2025 in the EU. FMD is endemic in some countries in the Mediterranean basin as well as Turkey, where in addition to historically present serotypes (A, O and Asia1), SAT2 has been recently introduced, and is currently spreading [3].

Preparedness is particularly important when FMD is introduced into a naive population, in areas densely populated with animals of susceptible species, where rapid response is essential to control its spread. A study of a hypothetical FMD outbreak in California concluded that delaying outbreak response from 7 days to 22 days increases the estimated economic impact of the disease from $3 billion to $69 billion [4].

In the event the disease is introduced into the EU, FMD control strategies require the infection be rapidly eradicated by culling and destroying infected and suspected infected animals, so as to quickly regain disease-free status [5]. The EU legislation also provides for emergency vaccination in certain risk situations, especially in areas with a high density of animals of susceptible species [6].

Given the transmissibility characteristics of FMD, in order to speed up the eradication process, the legislation in force in the EU requires that some control activities be initiated in peacetime (non-epidemic period): this is the “emergency preparedness”, which is a component of national contingency plans. Commonly, a uniform national control policy is not always the best strategy to control diseases and high-risk regions should be treated in a different way from low-risk regions [7].

In Italy, different zoo technical realities coexist. In the north and centre of the country, intensive breeding prevails and in some areas and production sectors, such as pig farming, a high level of specialisation is achieved through integrated production circuits whereas in the remaining central and southern regions, family-run farms prevail; they are less specialised and have “mixed” breeding systems (pigs and cattle, cattle, sheep and goats, etc.). These elements must all be considered when preparing national contingency plans.

In the framework of a research project on FMD, a simulation model was developed to verify the effectiveness of the main strategies to control the spread of the disease in different livestock contexts, in accordance with the provisions laid down in Regulation (EU) 2016/429 [8] of the European Parliament and of the Council and Commission Delegated Regulation (EU) 2020/687 (Appendix A) [5]. For this purpose, three study areas with different zoo technical characteristics and animal density were chosen: (1) medium density, (2) high density, (3) an extensive livestock production area [9]. The classification of the areas was based on the method developed in an EU research project on FMD and classical swine fever (CSF), where animal density was calculated from the number of animals and the area of the municipality [10].

The results of the study conducted in Italy showed that in areas with low and medium density of FMD susceptible animals, the basic control measures required by current legislation (stamping out) were sufficient to control the infection, whereas in the dense areas of northern Italy, the control strategy based on the culling of outbreak animals was not sufficient to halt the spread of the infection and it was necessary to combine culling of outbreaks with emergency vaccination [9]. This confirmed that in non-endemic areas, emergency vaccination should be used as a complement to, rather than a replacement of, the culling of infected and at-risk farms [11].

This study focuses on the spread of FMD in densely populated areas of northern Italy, with the aim of characterizing these territories and identifying suitable and sustainable control measures to minimize the impact of the disease in the event of its introduction.

We simulated the reduction in FMD-susceptible population in the highest-risk area and its effect on the spread of the infection to identify threshold densities that could allow the eradication of the infection by applying only basic control measures, such as the culling of infected and at-risk farms. In a risk-based preventive reduction in farm density, we identified farms at the greatest risk of FMDV transmission using a typical network theory parameter, the degree of connectedness, which identified the farms most connected to other farms, taking into account the distance between farms as well as the farm’s susceptibility and infectivity.

## 2. Materials and Methods

### 2.1. Study Area

The study was conducted in Lombardy (northern Italy), one the areas with the highest livestock density in Italy. The region has an important position as it is the largest livestock producer in the EU in terms of pig and cattle breeding and processing industry (pork, milk).

**Figure 1:**
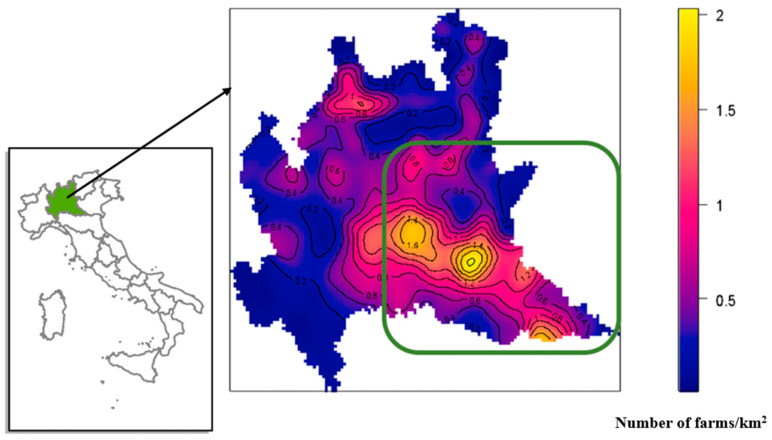
Lombardy: density of farms with more than 10 heads. The green box represents the study area.

Figure 1 shows the density of farms per km^2^ in Lombardy. For the purposes of this study, the area with the highest density of FMD-susceptible animals in the region was chosen. This area is between the provinces of Brescia, Mantua and Cremona and is enclosed in the green box.

Information on farms of FMD-susceptible animals was mined from the Italian National Database (NDB). The attributes considered in the study were the type of farm, the size of the farm, understood as the number of heads, and its geographical location.

To simplify the processing of data, this study considered farms with more than 10 heads and the municipality as the territorial reference unit. Table 1 shows details at a provincial level. In Italy, municipalities are grouped into provinces and provinces are grouped into regions.

For the classification of zones, we have taken an approach like the one developed by R.B. Huirne for FMD in 2023, which was based on the density of FMD susceptible animals per km^2^ [10]. The municipalities with fewer than 150 animals (dairy cows, beef cattle, sheep, goat and pigs)/km^2^ were classified as sparsely populated livestock areas (SPLAs). Those with between 151 and 450 animals were classified as medium-populated livestock areas (MPLAs), and those with more than 450 animals (or more than 300 pigs/km^2^) were classified as densely populated livestock areas (DPLAs).

However, given the Italian livestock system, where the number of animals on farms can vary throughout the year, we deemed it prudent to use the threshold of 400 animals per km^2^, rather than 450, for the DPLAs.

### 2.2. Characteristics of the Model

The population density of susceptible animals plays a crucial role in determining the evolution of an epidemic and this is particularly true for Transboundary Animal Diseases (TADs) such as avian influenza [12] or FMD [12,13]. The model was developed based on a dynamic network of transmission where local transmission of the infection is a function of the distance between farms, the infectivity and susceptibility of the farmed species and the type of farm. We adopted an approach similar to the one developed by the European Food Safety Authority (EFSA) to assess the effectiveness of control measures of the category A diseases in the Animal Health Law, including FMD [14] and in previous studies in the UK and Japan [15]. The probability of potentially infectious FMD contact between holdings accounts for a kernel of distance between farms, infectivity, and susceptibility of the animal species farmed and the herd size (Tables S1 and S2), as described by Pesciaroli et al. in 2025 [9]. Briefly, the component of probability of an infectious contact due to distance, assuming values between 0 and 1, was modelled by means of a kernel function, where we modified parameters from Bradhurst et al., 2022 [16]. The effect of animal species, type of production, size of the herd on the farm’s infectivity and susceptibility, and the associated uncertainty were modelled by means of a Beta distribution. As shown in Pesciaroli et al. 2025 [9], susceptibility was greatest for cattle and buffalo farms, whereas infectivity was greatest for large pig farms. The combination of such parameters contributed to the probability of infectious contacts between farms. Finally, an adjacency matrix was obtained based upon the probability of infection p(a) resulting from the combination of the distance kernel, and farms’ susceptibility and infectivity: a random number between 0 and 1 was extracted; if its value was above the value of p(a), the arc was created and the infection can be transmitted. For each simulation run, the probability of transmission was recalculated and a new network was created, resulting in a dynamic network that changed over time. The dynamic network was created by running 1000 simulations [9].

The data were processed in order to obtain an adjacency matrix between farms and establish a probability value of a potentially infectious contact to occur between pairs of farms based on geographical distance.

**Figure 2:**
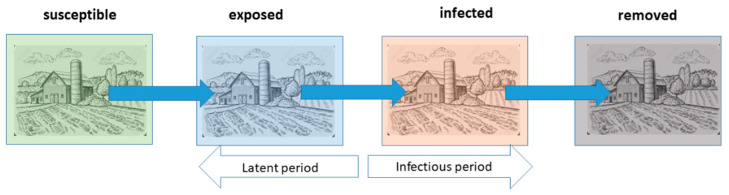
SEIR compartmental model. The SEIR model divides the population into four categories, called “S = Susceptible”, “E = Exposed”, “I = Infected”, and “R = Removed”.

Starting from the adjacency matrix, a subsequent 1000 simulations were carried out using the “Ndlib” library in Python (version 3.11.5) [17] which, from the adjacency matrix developed in R (version 4.4.2) [18], applied a SEIR compartmental model (Figure 2).

For each day after the simulation began, the number of infected farms was obtained as well as the median, first and third quartiles, and 95th percentile of outbreaks were calculated [9].

The high-risk area of northern Italy was initially based on the identified methodology developed by Huirne in 2003 [10]. In this study, we verified whether the risk threshold previously identified based on a density distribution could be confirmed using the dynamic transmission model developed in this study.

To calculate a threshold in terms of density of head/km^2^ that was compatible with controlling the disease without resorting to emergency vaccination, infection transmission simulations were conducted by randomly removing 500, 750, 1000, 1500, 2000, 2300, 2400 and 2500 farms from the study area (green box in Figure 1) and for each day the median, first and third quartiles, and 95th percentile of infected farms were estimated.

The simulations identified the three municipalities most involved in the spread of the infection due to the proximity of farms and the concentration of animals. Therefore, to simulate the worst-case scenario, the same analysis was conducted by reducing the number of farms in the municipalities ranked as the highest infection risk; those most frequently singled out during the simulations. It was thus possible to estimate the weighted density of heads/km^2^ and the number of infected herds for each day since the start of the simulation and results were expressed as median, first and third quartiles, and 95th percentile.

Subsequently, to improve the effectiveness of the population reduction in containing the spread of the infection, the analysis was conducted by eliminating only the “most interconnected” farms, i.e., those identified most frequently during the simulations. Each simulation generated the node degree for each farm; it expressed the number of edges adjacent to the node which was calculated using the *DiGraph.degree* function in the *NetworkX* library in Python (version 3.11.5). The assessment of the node degree allowed identifying and removing farms that triggered the greatest number of potentially infecting contacts. The simulation was repeated by removing these target farms to obtain a population density compatible with disease control [19]. The parameters of the model were specified in Tables S1 and S2 of the Supplementary Materials. Preliminary data manipulations, statistical analyses of modelling results, adjacency matrices, and maps were performed using R software.

### 2.3. FMD Control Strategies

The original project considered the main FMD control strategies provided by current legislation [5], and, in particular, the following:

A. culling of animals from infected farms (baseline scenario, stamping out),

B. culling of outbreaks and preventive depopulation of susceptible animals within a maximum buffer zone of 5 km from the outbreak,

C. culling of outbreak animals and emergency vaccination.

As reported in the introduction, the first two strategies were unable to interrupt the infection transmission chain in DPLAs.

## 3. Results

The study area between the provinces of Brescia, Mantua, and Cremona was 3020.7 km^2^, the number of FMD-susceptible species farms was 3074, whereas the total number of heads was 2,836,649 with an average density of 940 heads/km^2^ (min 4.51–max 2147.13 heads/km^2^).

In this area, the average density of farms was approximately 0.74 farms per km^2^, with the sub-area having a maximum density of approximately two farms per km^2^ (Figure 1, in the green box).

The maximum density of animals was in the municipality of Gottolengo, with 2147.13 heads/km^2^, followed by Calvisano (194,590 heads/km^2^), and Acquafredda (1143.39 heads/km^2^) (Table 2). These municipalities can be considered hyper-dense and due to their concentration of animals, it is likely that it would not be operationally simple to promptly intervene in the case of FMDV introduction.

To identify the density of animals compatible with the interruption of the transmission chain and to compare it with that previously identified by Huirne [10], 500 farms were randomly removed through 1000 simulations. The results of these simulations showed that by eliminating 500 farms, the average density of heads/km^2^ in the remaining 2574 farms was 785.27 heads/km^2^, which was not effective in interrupting the transmission of the infection. Hence, to identify the threshold, the same procedure was used to randomly remove 750, 1000, 1500, 2000, 2300, 2400, and 2500 farms, and for each of them the density of animals/km^2^ was calculated (Table 3).

**Figure 3:**
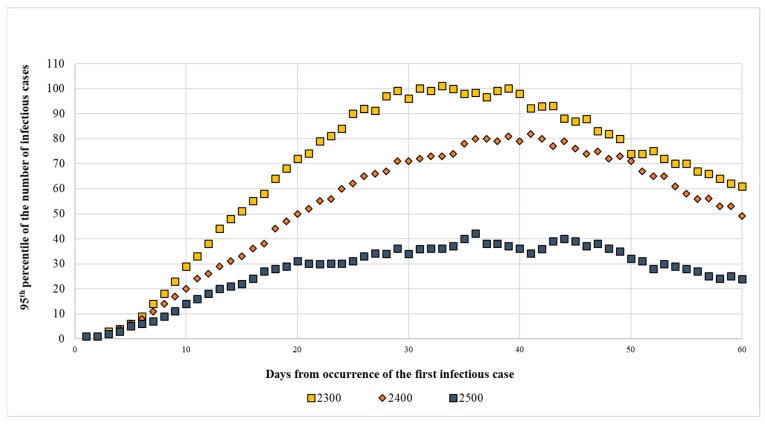
Graphical representation of a two-month FMD epidemic in the area of the study. The graph compares the effects of removing 2300, 2400, and 2500 farms; the results reported are those of 95th percentile.

The figure below shows the detailed progress of the three scenarios requiring 2300, 2400, and 2500 farms to be randomly removed from the study area as they began to provide results compatible with controlling the infection (Figure 3). The complete simulation results are reported in the Supplementary Materials (Tables S3–S5).

By removing 2300 farms, the weighted density of heads/km^2^ was 473.60, which represented 74.8% of the farms present in the three municipalities. If 2400 farms were removed, the density was 412.41 heads/km^2^, which was obtained by removing 78.1% of the farms. Removing 2500 farms gave a density of 351.22 heads/km^2^ which implied removing 81.3% of farms. The density calculations in the three municipalities at the highest risk are reported in Table 4.

For the sake of completeness, the results of 60 days of simulation are reported and for each day, the median of the number of outbreaks, the third quartile and the 95th percentile are shown; the data reported are the result of 1000 simulations. The complete results are reported in the Supplementary Materials.

In summary, the analysis showed the following:

- Applying a threshold density of 473.60 heads/km^2^, considering the median, the epidemic ended on the 15th day. Instead, if the third quartile was considered, the epidemic would end on the 37th day with 64 outbreaks. However, in 5% of cases, on the same date there would be almost 100 outbreaks of which 61 could still be present on the 60th day of simulation (Table S3).
- Applying a density of 412.41 heads/km^2^, considering the median, the epidemic ended on the 15th day. Instead, if the third quartile was considered, the epidemic would end on the 36th day with 50 outbreaks. In 5% of cases, 49 outbreaks could still occur on the 60th day (Table S4).
- Applying a density of 351.22 heads/km^2^, considering the median, the epidemic ended on the 13th day. If the third quartile was considered, the epidemic would end on the 30th day with 36 outbreaks. In 5% of cases, on the 60th day there are still 24 outbreaks (Table S5).

Based on the simulation results, the density of head/km^2^ compatible with disease control seemed to be between 412 and 351 heads/km^2^, even if some outbreaks continue to occur. However, it was considered that a certain level of risk must be accepted and must be managed by applying adequate prevention and control measures. Indeed, to achieve more effective control of the disease, a density of 351.22 heads/km^2^ should be reached, which would be achieved by drastically eliminating 81.3% of existing farms.

Furthermore, the analysis of the contact networks showed that the farm playing the greatest role in the spread of the infection was found 615 times (61.5%) in the municipality of Calvisano, 137 times in the municipality of Gottolengo and 102 times in the municipality of Acquafredda. These three municipalities are mutually neighbouring and were identified in 85.4% of the simulations.

Nevertheless, the study was repeated in the same area, selectively eliminating the farms with the highest risk potential, the most “interconnected”. Applying this approach, the control action was understandably more effective: the removal of 500 target farms was more effective in controlling the epidemic than the random removal of 1000 farms (Figure S1). This part of the study was not aimed at identifying the threshold density but rather at understanding the potential impact of farms with a higher level of risk on the development of the epidemic. Indeed, for preparedness purposes, it would be advisable to identify these farms during non-epidemic periods to adopt appropriate preventive measures, such as strengthened surveillance and rigorous biosecurity measures, in order to mitigate their risk of spreading the infection.

## 4. Discussion

Factors determining the spread of an infection include the frequency and type of contact between farms in the period following infection, before the infection is detected and before the farm is isolated, as well as the density of farms in the area surrounding the infected farm. These factors play a pivotal role in the development of an epidemic, especially when the pathogen is introduced into a densely populated area.

A recent research project conducted in Italy showed that in areas with low and medium density of a susceptible species, FMD outbreaks can be managed using the basic control measures provided by the current legislation on FMD [9] which is based on the strategy of killing the infected herds (stamping out) and any animal in contact with them coupled with appropriate disposal or management of potentially infective animal products, such as food products, animal by-products, persons, vehicles, farm fomites, and any other substance liable to transmit the virus [5].

Differently, in the Italian DPLA, the control strategy based on the culling of outbreak animals and ring vaccination was the most cost-effective [9].

For the classification of the territories, we adopted an approach similar to the one developed by an EU-funded project in the 2000s, where a method was established to identify high risk areas in the EU; some areas of Northern Italy were classified by this study as densely populated. The classification of the areas was based on the animal density per km^2^ and 5% of the municipalities with the highest density (over 450 animals per km^2^) were those to be considered at highest risk of transmission (DPLAs). Based on their assumptions, if FMDV were introduced in these areas, additional control measures, such as preventive culling of animals and/or emergency vaccination, would have been necessary to achieve eradication [10].

The approach adopted had the advantage of being simple and easily applicable in all EU countries. However, despite the simplicity of the approach, it was noted that these areas were very often heterogeneous, with some sub-areas being highly dense and others less dense. Therefore, for prevention and control purposes, it would have been relevant to characterize their spatial structure and livestock production to be able to specifically target control activities.

The study reported in this article was focused on characterizing the DPLAs in Lombardy and verifying whether the risk threshold previously identified based on a density distribution could be confirmed using the dynamic transmission model developed in the current study. The threshold to establish the areas at highest risk of transmission obtained in this study is between 412 and 351 heads/km^2^, not very different from that identified in the previous study.

Sub-areas where the density of susceptible animals greatly exceeded the effective threshold for disease control were identified in northern Italy and through the analysis of the contact networks, it was found that the farms playing the greatest role in the spread of the infection were detected in 85.4% of the simulations in three contiguous municipalities: Gottolengo, with 2147.13 heads/km^2^, followed by Calvisano (1945.90 heads/km^2^) and Acquafredda (1143.39 heads/km^2^). In these sub-areas, it would most likely be difficult to manage FMD even if emergency vaccination was carried out, considering that simultaneously the animals from the outbreaks would have to be slaughtered and disposed of.

Therefore, for preparedness purposes, it is necessary to carefully evaluate the capacity to dispose of carcasses and, if necessary, identify alternative solutions. It is important to remember that culling animals requires significant human resources and that carcass disposal can represent a bottleneck that hinders the completion of the eradication strategy.

Contingency plans for some epidemic diseases, such as highly pathogenic avian influenza, encourage the adoption of measures aimed at reducing the density of susceptible animals in areas at high risk of transmission to facilitate intervention in the event of disease introduction.

This would be the situation in the study area if 1760 farms, i.e., 57.2% of existing farms were removed (Table 3). While this approach could prove effective in containing the spread of the infection, it would certainly be unpopular with stakeholders and citizens, and it would be difficult to accept in both economic and ethical terms.

However, in our study, contact network analysis allowed us to identify farms that play a crucial role in the spread of the infection. Therefore, the study was repeated in the same area, selectively eliminating the farms with the highest risk of transmission, i.e., those most “interconnected”. This simulation aimed at determining the impact of these farms on the development of an epidemic. The application of this targeted approach made the control action more effective; indeed, the removal of 500 targeted farms was more effective in controlling the epidemic than the random removal of 1000 farms (Figure S1). In the light of preparedness, these high of-risk farms should be subjected to control measures proportionate to the risk they pose, in terms of animal movement control, biosecurity, and surveillance aimed at early detecting the incursion of the infection [20].

The model developed in this study proved suitable for planning the actions envisaged in contingency plans, characterizing areas at risk of FMD spread, identifying the most appropriate control strategies, verifying risk thresholds, and identifying farms at the highest risk of FMD transmission. These are all emergency preparatory actions intended to facilitate decision-making in the event of FMDV introduction. In our models, we included inter-farm distance and farm type (herd size and animal species) among factors affecting the probability of FMD virus transmission, in line with other disease control models developed in Europe and Asia [15]. This was justified by the analysis of previous FMD epidemics, and by the objective of the study. Furthermore, to attain an original risk-based approach to guide farm removal, we used the degree as a typical network parameter. This allowed us to identify the most connected farms, those most likely to play a central role in FMD virus transmission. This approach allows for more efficient disease control and can be applied in practical applications where limited information on inter-farm contact is available.

Further development of mathematical models includes the analysis of more detailed information, such as infection transmission routes and the effects of biosecurity measures. Models with these characteristics are currently being studied internationally.

## 5. Conclusions

Contingency plans have proven to be crucial tools for the successful control of epidemic diseases and modelling their spread lays the foundation for the design of a sound contingency plan. However, it is useful to remember that disease spread models are a simplification of more complex systems, they can be realistic but do not faithfully represent reality and consequently lead to generalized results.

In the light of preparedness, it is worth remembering that the key elements indicating that a culling policy alone is insufficient to halt the spread of the infection are, firstly, the number of outbreaks and the rapidity with which the disease spreads. Whatever the circumstances, the decision to use vaccination should be made very rapidly, from a few days to a week after the disease is detected [21].

In this regard, the time to detect the primary outbreak remains crucial for choosing the appropriate control method. Indeed, research conducted by H. Yoon [22] showed that, compared to all the other control strategies assessed in the study, reducing the number of days from disease incursion to commencement of control had the greatest impact on the epidemic size. Applying controls five days earlier resulted in shorter epidemics and a significantly smaller number of infected and depopulated farms. On the contrary, increasing the number of days from incursion to control resulted in larger and more variable epidemics [22]. Therefore, in non-epidemic period, all the activities aimed at enhancing early detection, such as training, awareness campaigns, target surveillance, etc., should be promoted.

## Figures and Tables

**Table 1:** Farms with FMD susceptible animals in the provinces of Brescia, Mantua and Cremona (green box).

Province	Nr. Farms	Nr. Animals
Brescia	1349	1,228,437
Cremona	424	479,812
Mantua	1301	1,128,400
Total	3074	2,836,649

**Table 2:** Details of the three municipalities on which the analysis focused, since they were identified in 85.4% of the simulation.

Municipality	Nr. Farms	Nr. Farms (>10 Animals)	Nr. Animals	Nr. Animals (Farms with >10 Animals)	Area	Density	Density (Farms with >10 Animals)
Acquafredda	12	7	10,916	10,915	9.55	1143.39	1143.29
Calvisano	173	100	87,236	87,149	44.83	1945.90	1943.96
Gottolengo	99	58	62,898	62,881	29.29	2147.71	2147.13
Total	284	165	161,050	160,945			

**Table 3:** Number of farms “removed”, percentage of farms “removed” calculated on 3074 farms included in analysis and the density of heads/km^2^ without farms “removed”.

Nr. Farms “Removed”	% Farms “Removed”	Density (Animals/km^2^)
0	0.0%	939.08
500	16.3%	785.27
750	24.4%	708.97
1000	32.5%	633.21
1500	48.8%	480.02
2000	65.1%	327.20
2300	74.8%	235.64
2400	78.1%	205.18
2500	81.3%	174.91

**Table 4:** Calculation of the weighted densities of the three municipalities on which the analysis was focused using the results obtained with 3074 farms.

Nr. Farms “Removed” (from 3074)	% Farms “Removed”	Nr. Farms (>10 Animals) “Removed”	Nr. Farms (>10 Animals) “Removed” (from 165)	Density (Animals/km^2^) Weighted *
2300	74.8%	123.4	41.6	473.60
2400	78.1%	128.9	36.1	412.41
2500	81.3%	134.1	30.9	351.22

* Weights were calculated considering the role of each municipality in the spread of the infection. (Acquafredda 102/854 times, Calvisano 615/854 times and Gottolengo 137/854 times).

## Data Availability

The original contributions presented in this study are included in the article/Supplementary Materials. Further inquiries can be directed to the corresponding author(s).

## Supplementary Materials

The following supporting information can be downloaded at https://www.mdpi.com/article/10.3390/pathogens14090933/s1, Table S1. Susceptibility and Infectivity parameters of the farm types. Table S2. Transmission parameters used in the SEIR model. Table S3. Median, third quartile, and 95th percentile of the number of FMDV infected farms, for 60 days from the first outbreak, calculated on the results of 1000 simulations using a computational dynamic model, after the random removal of 2300 farms, during each simulation, from a high-density study area including 3074 farms. The first infected case was identified causally, for each simulation, among the farms remaining after removal. Table S4. Median, third quartile, and 95th percentile of the number of FMDV infected farms, for 60 days from the first outbreak, calculated on the results of 1000 simulations using a computational dynamic model, after the random removal of 2400 farms, during each simulation, from a high-density study area including 3074 farms. The first infected case was identified causally, for each simulation, among the farms remaining after removal. Table S5. Median, third quartile, and 95th percentile of the number of FMDV infected farms, for 60 days from the first outbreak, calculated on the results of 1000 simulations using a computational dynamic model, after the random removal of 2500 farms, during each simulation, from a high-density study area including 3074 farms. The first infected case was identified causally, for each simulation, among the farms remaining after removal. Figure S1. Effect of removal of target farms on epidemic progression compared to removal of random farms. Figure S2. Effect of targeted removal on FMD transmission dynamics.

## Abbreviations

The following abbreviations are used in this manuscript:

FMD	Foot-and-Mouth Disease
DPLAs	Densely Populated Livestock Areas
TADs	Transboundary Animal Diseases

## Appendix A

Commission delegated regulation (EU) 2023/361 of 28 November 2022 supplementing Regulation (EU) 2016/429 of the European Parliament and the Council as regards rules for the use of certain veterinary medicinal products for the purpose of prevention and control of certain listed diseases.
**PART II:** rules on the use of vaccines for the prevention and control of category A diseases in animals.
**PART 2**
**SIMPLIFIED ASSESSMENT OF THE VACCINATION STRATEGY**
1. Number of establishments where the category A disease has been confirmed or suspected;
2. Type of establishments where the category A disease has been confirmed or suspected;
3. Number of animals kept in the establishments where the category A disease has been confirmed or suspected;
4. Species affected;
5. Capacity of killing and disposal schedule in establishments where animals are killed;
6. Rate of airborne or vector-borne spread of the disease agent from the establishments or area where the category A disease has been confirmed.
