# Supplementary material for: Simulating the Spread of Foot-and-Mouth Disease in Densely Populated Areas as Part of Contingency Plans to Establish the Best Control Options
Source: Pathogens. 2025 Sep 16;14(9):933. doi: 10.3390/pathogens14090933 (PMC12472710; doi:10.3390/pathogens14090933)
Supplement: Supplementary file 1 [file pathogens-14-00933-s001.zip › pathogens-3807703-supplementary.pdf]

Supplementary Materials

Table S1: Susceptibility and Infectivity parameters of the farms type.

Farm	Susceptibility	Infectivity
Large dairy bovine	Beta (90, 10)	Beta (45, 55)
Large cattle	Beta (90, 10)	Beta (45, 55)
Small size bovine	Beta (90, 10)	Beta (25, 75)
Water buffalo	Beta (90, 10)	Beta (45, 55)
Small ruminants	Beta (15, 85)	Beta (25, 75)
Large swine fattening	Beta (6, 94)	Beta (90, 10)
Large swine breeders	Beta (6, 94)	Beta (90, 10)
Small swine	Beta (6, 94)	Beta (45, 55)
Back-yard	Beta (5, 95)	Beta (5, 95)

Table S2: Transmission parameters used in the SEIR model.

Model Parameter	Parameter	Value
Transmission Rate between Infectious to Susceptible	β	0.25
Transition rate between Exposed to Infected	$1/\alpha$	0.5
Transition Rate between Infected to Removed or Removal Rate of Infectious	$1/\gamma$	0.1

Table S3. Median, third quartile, 95th percentile of the number of FMDV infected farms, for 60 days from the first outbreak, calculated on the results of 1000 simulations using a computational dynamic model, after the random removal of **2300** farms, during each simulation, from a high-density study area including 3074 farms. The first infected case was identified causally, for each simulation, among the farms remaining after removal.

**Day**	**1**	**2**	**3**	**4**	**5**	**6**	**7**	**8**	**9**	**10**	**11**	**12**	**13**	**14**	**15**	**16**	**17**	**18**	**19**	**20**
**Median**	1	1	1	1	1	1	1	1	1	1	1	1	1	1	0	0	0	0	0	0
**3rd quartile**	1	1	1	1	2	2	2	2	2	2	3	3	3	3	3	3	3	3	2	2
**95th percentile**	1.00	1.00	3.00	4.00	6.00	9.05	14.05	18.05	23.00	29.05	33.05	38.05	44.00	48.05	51.05	55.10	58.05	64.05	68.05	72.10

**Day**	**21**	**22**	**23**	**24**	**25**	**26**	**27**	**28**	**29**	**30**	**31**	**32**	**33**	**34**	**35**	**36**	**37**	**38**	**39**	**40**
**Median**	0	0	0	0	0	0	0	0	0	0	0	0	0	0	0	0	0	0	0	0
**3rd quartile**	2	2	2	2	1	1	1	1	1	1	1	1	1	1	1	1	0	0	0	0
**95th percentile**	74.10	79.05	81.05	84.10	90.05	92.05	91.30	97.05	99.10	96.15	100.10	99.10	101.05	100.00	98.00	98.50	96.60	99.15	**100.15**	98.05

**Day**	**41**	**42**	**43**	**44**	**45**	**46**	**47**	**48**	**49**	**50**	**51**	**52**	**53**	**54**	**55**	**56**	**57**	**58**	**59**	**60**
**Median**	0	0	0	0	0	0	0	0	0	0	0	0	0	0	0	0	0	0	0	0
**3rd quartile**	0	0	0	0	0	0	0	0	0	0	0	0	0	0	0	0	0	0	0	0
**95th percentile**	92.25	93.05	93.10	88.15	87.05	88.00	83.10	82.00	80.00	74.05	74.05	75.05	72.05	70.05	70.00	67.05	66.00	64.05	62.05	61.00

Table S4. Median, third quartile, 95th percentile of the number of FMDV infected farms, for 60 days from the first outbreak, calculated on the results of 1000 simulations using a computational dynamic model, after the random removal of **2400** farms, during each simulation, from a high-density study area including 3074 farms. The first infected case was identified causally, for each simulation, among the farms remaining after removal.

Day	1	2	3	4	5	6	7	8	9	10	11	12	13	14	15	16	17	18	19	20
Median	1	1	1	1	1	1	1	1	1	1	1	1	1	1	0	0	0	0	0	0
3rd quartile	1	1	1	1	2	2	2	2	2	2	2	2	2	2	2	2	2	2	2	1
95th percentile	1.00	1.00	2.00	4.00	6.00	8.00	11.00	14.00	17.00	20.00	24.05	26.00	29.05	31.00	33.00	36.05	38.10	44.05	47.05	50.00

Day	21	22	23	24	25	26	27	28	29	30	31	32	33	34	35	36	37	38	39	40
Median	0	0	0	0	0	0	0	0	0	0	0	0	0	0	0	0	0	0	0	0
3rd quartile	1	1	1	1	1	1	1	1	1	1	1	1	1	1	1	0	0	0	0	0
95th percentile	52.05	55.05	56.00	60.05	62.10	65.10	66.05	67.00	71.00	71.05	72.05	73.05	73.05	74.05	78.00	80.05	80.00	79.00	81.00	79.00

Day	41	42	43	44	45	46	47	48	49	50	51	52	53	54	55	56	57	58	59	60
Median	0	0	0	0	0	0	0	0	0	0	0	0	0	0	0	0	0	0	0	0
3rd quartile	0	0	0	0	0	0	0	0	0	0	0	0	0	0	0	0	0	0	0	0
95th percentile	82.00	80.05	77.00	79.05	76	74	75	72.05	73	71	67	65	65	61	58	56	56.05	53.10	53.00	49.05

Table S5. Median, third quartile, 95th percentile of the number of FMDV infected farms, for 60 days from the first outbreak, calculated on the results of 1000 simulations using a computational dynamic model, after the random removal of **2500** farms, during each simulation, from a high-density study area including 3074 farms. The first infected case was identified causally, for each simulation, among the farms remaining after removal.

Day	1	2	3	4	5	6	7	8	9	10	11	12	13	14	15	16	17	18	19	20
Median	1	1	1	1	1	1	1	1	1	1	1	1	0	0	0	0	0	0	0	0
3rd quartile	1	1	1	1	1	1	2	2	2	2	2	2	2	2	1	1	1	1	1	1
95th percentile	1.00	1.00	2.00	3.00	5.00	6.00	7.00	9.00	11.01	14.00	16.00	18.05	20.05	21.05	22.05	24.05	27.05	28.05	29.00	31.00

Day	21	22	23	24	25	26	27	28	29	30	31	32	33	34	35	36	37	38	39	40
Median	0	0	0	0	0	0	0	0	0	0	0	0	0	0	0	0	0	0	0	0
3rd quartile	1	1	1	1	1	1	1	1	0.25	0	0	0	0	0	0	0	0	0	0	0
95th percentile	30.05	30.00	30.10	30.10	31.00	33.05	34.10	34.05	36.10	34.05	36.00	36.10	36.10	37.15	40.00	42.05	38.15	38.10	37.05	36.05

Day	41	42	43	44	45	46	47	48	49	50	51	52	53	54	55	56	57	58	59	60
Median	0	0	0	0	0	0	0	0	0	0	0	0	0	0	0	0	0	0	0	0
3rd quartile	0	0	0	0	0	0	0	0	0	0	0	0	0	0	0	0	0	0	0	0
95th percentile	34.20	36.00	39.00	40.05	39.05	37.05	38.05	36.05	35.05	32.15	31.05	28.05	30	29	28	27	25.10	24.10	25.05	24.00

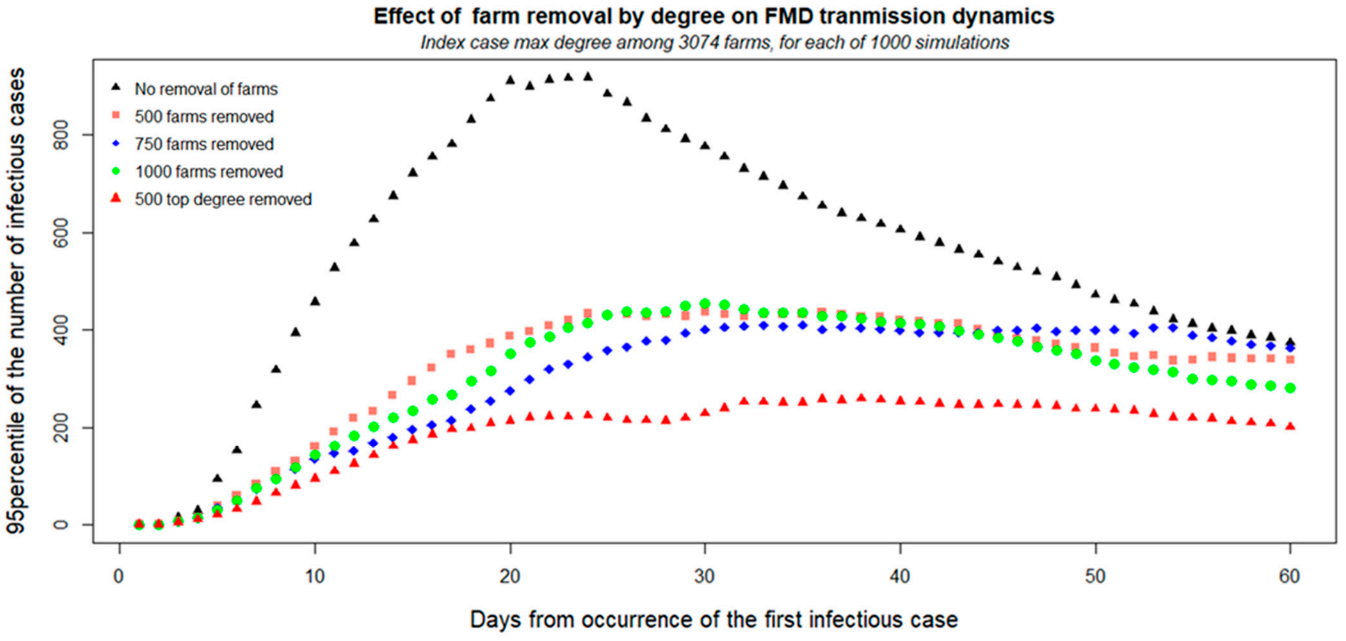

Figure S1. Effect of removal of target farms on epidemic progression compared to removal of random farms.

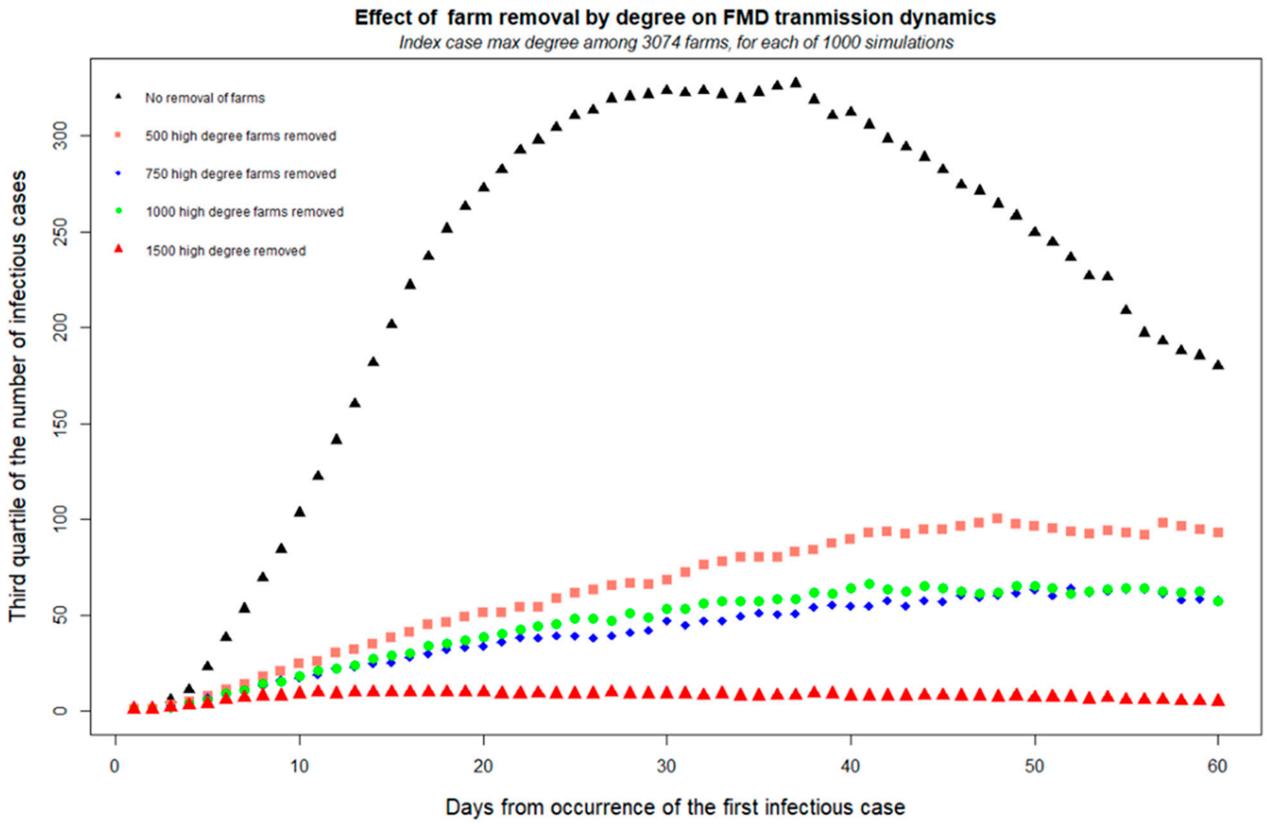

Figure S2. Effect of Targeted Removal on FMD transmission dynamics
